# A Novel Locally c-di-GMP-Controlled Exopolysaccharide Synthase Required for Bacteriophage N4 Infection of *Escherichia coli*

**DOI:** 10.1128/mbio.03249-21

**Published:** 2021-12-14

**Authors:** Eike H. Junkermeier, Regine Hengge

**Affiliations:** a Institut für Biologie/Mikrobiologie, Humboldt-Universität zu Berlin, Berlin, Germany; Institut Pasteur

**Keywords:** nucleotide second messenger, c-di-GMP, diguanylate cyclase, DgcJ, glycosyltransferase, enterobacterial common antigen, ManNAc, bacteriophage N4, biofilm, exopolysaccharide

## Abstract

A major target of c-di-GMP signaling is the production of biofilm-associated extracellular polymeric substances (EPS), which in Escherichia coli K-12 include amyloid curli fibers, phosphoethanolamine-modified cellulose, and poly-*N*-acetylglucosamine. However, the characterized c-di-GMP-binding effector systems are largely outnumbered by the 12 diguanylate cyclases (DGCs) and 13 phosphodiesterases (PDEs), which synthetize and degrade c-di-GMP, respectively. E. coli possesses a single protein with a potentially c-di-GMP-binding MshEN domain, NfrB, which—together with the outer membrane protein NfrA—is known to serve as a receptor system for phage N4. Here, we show that NfrB not only binds c-di-GMP with high affinity but, as a novel c-di-GMP-controlled glycosyltransferase, synthesizes a secreted EPS, which can impede motility and is required as an initial receptor for phage N4 infection. In addition, a systematic screening of the 12 DGCs of E. coli K-12 revealed that specifically DgcJ is required for the infection with phage N4 and interacts directly with NfrB. This is in line with local signaling models, where specific DGCs and/or PDEs form protein complexes with particular c-di-GMP effector/target systems. Our findings thus provide further evidence that intracellular signaling pathways, which all use the same diffusible second messenger, can act in parallel in a highly specific manner.

## INTRODUCTION

Many key cellular functions in bacteria, ranging from adhesion and biofilm formation to development and virulence, are controlled by the second messenger bis-(3′,5′)-cyclic-di-guanosine-monophosphate (c-di-GMP) (1). Remarkably, the genomes of most bacteria encode a multitude of diguanylate cyclases (DGCs) and phosphodiesterases (PDEs) that synthesize and degrade c-di-GMP, respectively (2). In Escherichia coli K-12, most of its 12 DGCs and 13 PDEs are not just expressed but are also active at the same time (3–5). This multiplicity raised the question of how c-di-GMP signaling can be specific, since all of the c-di-GMP-controlled effector/target systems rely on the same diffusible intracellular second messenger (6). Moreover, knockout mutations, in particular single DGCs or PDEs, were found to result in strong phenotypes without affecting the strikingly low intracellular c-di-GMP level in E. coli, which does not exceed 100 nM even in stationary-phase cells, i.e., when the characterized effector/target systems are clearly active (5). These seemingly enigmatic observations could be resolved by a model of “local signaling,” in which a master PDE (PdeH) maintains a very low global c-di-GMP pool, whereas specific DGCs, which are directly and locally associated with specific effector/target systems, can act as local and dynamic c-di-GMP sources to trigger specific responses. Similarly, specific PDEs associated with effector/target systems can act as local sinks of c-di-GMP and thus inhibit the regulatory output (recently reviewed in reference 7).

Prototypical examples of such locally c-di-GMP-controlled systems have been examined thoroughly in E. coli. For example, cellulose synthesis, modification, and secretion by the Bcs machinery not only is c-di-GMP controlled (8, 9) but also depends specifically on the diguanylate cyclase DgcC (YaiC) (10). By being directly localized to the core BcsAB complex via protein-protein interactions, DgcC and PdeK serve as a source and sink of c-di-GMP, respectively, for the c-di-GMP-binding PilZ domain of the cellulose synthase subunit BcsA (11). In this example, the main function of the protein-protein interactions is to colocalize the source and sink of c-di-GMP to its receptor binding site. In addition, protein-protein interactions between specific DGCs/PDEs and their respective effector/target systems can also assume regulatory functions. Thus, the expression of the biofilm regulator CsgD is controlled by the locally acting DgcE-PdeR-DgcM-MlrA signaling module. In this system, the “trigger PDE” PdeR directly binds and thereby inhibits DgcM and the transcription factor MlrA. When PdeR becomes active as a PDE, i.e., degrades c-di-GMP that is provided specifically by DgcE, this direct inhibition is released with two consequences: DgcM can produce c-di-GMP, which results in local positive feedback, and the transcription of *csgD* is initiated by the DgcM-MlrA complex (12–14).

**FIG 1:**
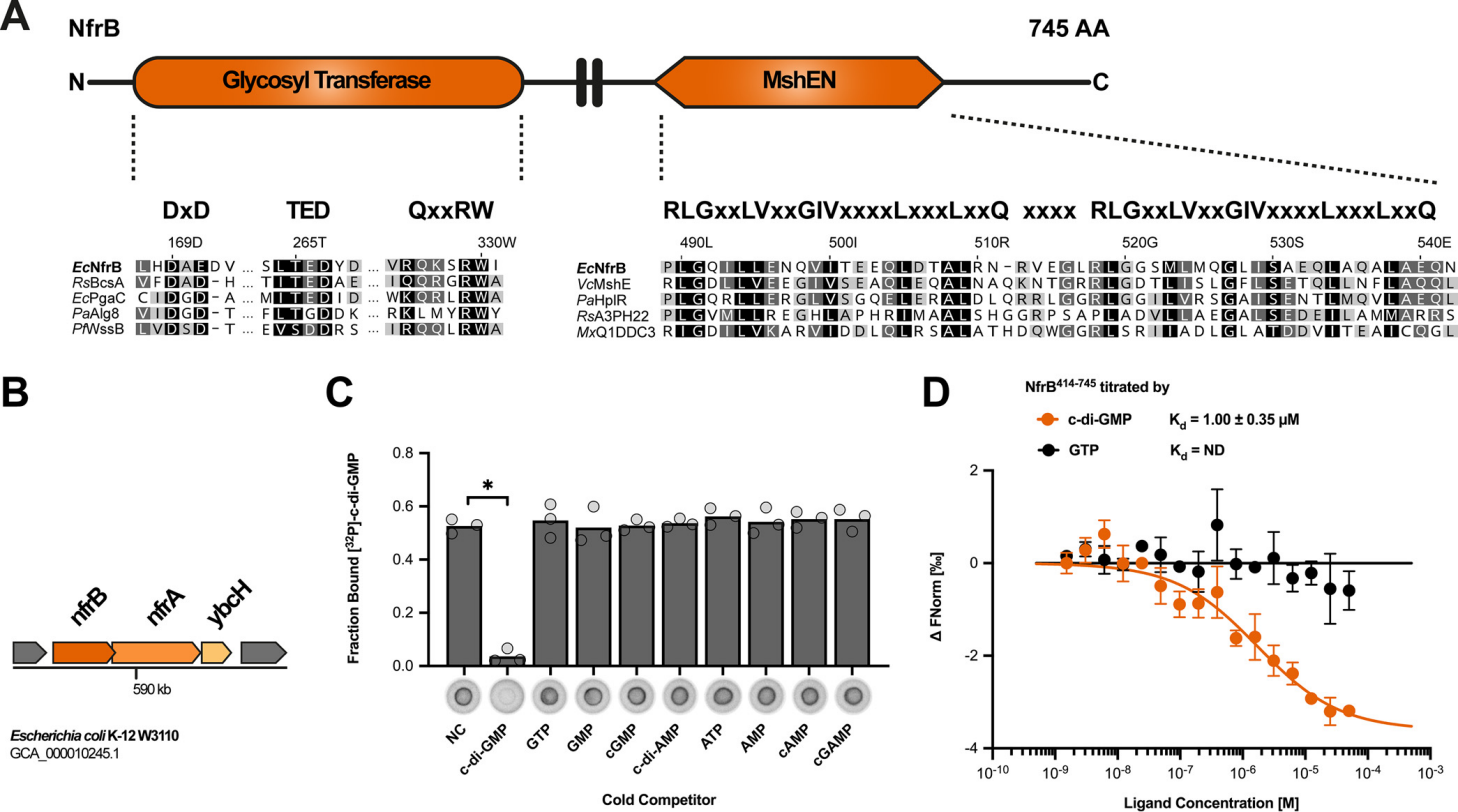
The MshEN domain of NfrB specifically binds c-di-GMP. (A) Domain structure of NfrB (top) and alignment of its glycosyltransferase domain and MshEN domain (bottom). Residues critical for glycosyltransferase activity (25) and binding of c-di-GMP (17) are highlighted above the alignment. (B) Organization of the *nfrBA-ybcH* operon in E. coli K-12. The *orf* of *nfrB* is overlapping by 14 nucleotides with the open reading frame of *nfrA*. (C) Differential radial capillary action of ligand assay (DRaCALA) of interactions between purified NfrB^414-745^ (20 μM) incubated with 4 nM ^32^P-labeled c-di-GMP. Excesses (500 μM) of unlabeled mono- and dinucleotides were added to the binding reactions, as indicated in the competition assays. Protein-ligand mixtures were spotted onto nitrocellulose and allowed to dry before imaging. Individual data points (cycles) and averages (bars) of the calculated fraction bound for three independent experiments are shown. Images of one representative competition assay are shown below the graph. Binding of ^32^P-labeled c-di-GMP is noticeable as dark spots centered on the nitrocellulose. NC, no competitor. *P* values below 0.001 are marked by an asterisk (*) and were assessed using a Student *t* test for significant differences compared to the NC control. (D) Interaction of NfrB^414-745^ with c-di-GMP and GTP was measured by microscale thermophoresis (MST). 40 nM labeled NfrB^414-745^ (RED-NHS dye; NanoTemper Technologies) was incubated with increasing concentrations (0.00153 to 50 μM) of c-di-GMP (*n* = 3) or GTP (*n* = 2) and measured by MST using Monolith NT.115 (NanoTemper Technologies) at 20% excitation power and 40% MST power. The change in normalized fluorescence (ΔFnorm in [‰]) is plotted against the concentration of the respective ligand. The dissociation constant (*K_d_*) was quantified by the *K_d_* fit of the NanoTemper Analysis software (v2.3).

Apart from these characterized systems, the considerable number of 12 DGCs and 13 PDEs of E. coli K-12—most of still unknown function—suggests the existence of additional c-di-GMP-controlled systems. In recent years, various approaches have led to the discovery of novel types of c-di-GMP-binding effector components in other bacteria. Among these, the N-terminal domain of MshE-type ATPases (termed MshEN domain), which are involved in type IV pilus formation of Vibrio cholerae, as well as bacterial type II secretion systems of Pseudomonas aeruginosa (15, 16), has been identified as a potent c-di-GMP binding receptor. The MshEN domain binds c-di-GMP in a unique fashion, in which a tandem array of two highly conserved 24-residue motifs (Fig. 1A) participates in extensive hydrophobic interactions with the dinucleotide (17). Bioinformatic studies revealed that the MshEN domain is a ubiquitous regulatory domain. Apart from being present in ATPases of type II and type IV secretion systems, it also seems to be involved in a variety of other bacterial processes, including two-component signaling, protein phosphorylation, polysaccharide secretion, and chemotaxis (17, 18). However, the large majority of these proteins have remained uncharacterized.

NfrB is the only protein containing the MshEN domain in E. coli. More than 30 years ago, NfrB was found to be part of a receptor system for bacteriophage N4, even though it is located in the inner membrane (19, 20). In addition, phage N4 infection requires the outer membrane protein NfrA, which is directly bound by the phage tail sheath protein gp65 (19–21). Moreover, the cytoplasmic protein NfrC (WecB), which also plays a role in the biosynthesis of the enterobacterial common antigen (ECA), has been implicated in phage N4 infection (22, 23).

Here, we show that E. coli NfrB is a c-di-GMP binding protein with a novel glycosyltransferase-MshEN domain architecture. We found that its ability to bind c-di-GMP and its glycosyltransferase active site are both essential for a successful phage N4 infection. We further provide evidence that the NfrB-NfrA system produces a novel, yet-uncharacterized exopolysaccharide that not only serves as an initial receptor for the phage N4 but that can also impede flagellar activity. Furthermore, successful phage N4 infection is shown to specifically require DgcJ, which directly contacts NfrB by protein-protein interaction, thus establishing a novel locally c-di-GMP-controlled system in E. coli. Starting from a very different angle, i.e., phage N4 biology, and using mainly genetics, the accompanying paper came to conclusions that are fully consistent with ours (24).

## RESULTS

### NfrB is a novel c-di-GMP-binding effector protein in *E. coli.*

Since the NfrB protein of E. coli K-12 is a larger protein with a fully conserved MshEN domain at its C terminus (Fig. 1A), we started our study by an analysis of its overall domain structure, as well as its genomic context. The N-terminal domain of NfrB shows similarity to family 2 glycosyltransferase (GT) and indeed features the conserved DxD, TED, and QxxRW active site signatures of processive GTs (25) (Fig. 1A). The two domains of NfrB are linked by two putative transmembrane helices, which most likely anchor NfrB in the inner membrane (see Fig. S1A). In E. coli, *nfrB* is encoded in an operon with *nfrA* and *ybcH* (Fig. 1B). A closer inspection of the NfrA protein sequence revealed that NfrA has a classical signal sequence, TPR-rich repeats, and a large C-terminal region that most probably forms an outer membrane pore (see Fig. S1C). YbcH is a hydrophilic protein with a N-terminal signal sequence-like region that lacks a cleavage site for signal peptidase, i.e., YbcH seems to be a periplasmic protein, which stays anchored in the inner membrane (see Fig. S1F). Taken together, the three proteins NfrB, NfrA, and YbcH show the key characteristics of a putative Gram-negative exopolysaccharide synthesis and secretion system, i.e., an inner membrane-located polysaccharide synthase (NfrB), a periplasmatic putative scaffold protein (YbcH), as well as an outer membrane TPR-containing β-barrel protein (NfrA).

10.1128/mbio.03249-21.1 FIG S1 The Nfr system shows the typical arrangement of a polysaccharide secretion system. (A) Structure prediction of NfrB (identifier AF-P0AFA5-F1) by AlphaFold v2.0 (53). The N-terminal domain (NfrB^1-450^) is displayed as a cartoon backbone representation. Helical parts are colored magenta, and beta sheet structures are colored yellow. Three predicted alpha-helices could serve as membrane anchors in the inner membrane. In this configuration, the active site signature (DxD, TED, and QxxRW) forms a putative open cavity for substrate binding. (B) Amino acid sequence of NfrA (UniProt identifier P31600). The signal sequence is highlighted in gray, and a putative cleavage site for the signal peptidase is marked by an arrowhead. Predicted TPR repeats (see panel D) are shown in green, and the predicted C-terminal beta barrel is marked in yellow (see panel C). (C) Structure prediction of NfrA (identifier AF-P31600-F1) by AlphaFold 2.0. A 12-stranded β-barrel (yellow) could serve as a pore for the secretion of an exopolysaccharide synthetized by NfrB. (D) Tetratricopeptide repeat motifs of NfrA as predicted by TPRpred (54) (https://toolkit.tuebingen.mpg.de/tools/tprpred). (E) YbcH (UniProt identifier P37325) lacks a cleavage site for the signal peptidase in the signal sequence (gray). (F) AlphaFold v2.0. predicts a TIM barrel-like structure (8-strand beta barrel surrounded by 7 α-helixes) for YbcH (AF-P37325-F1). YbcH most likely remains anchored in the inner membrane with its N terminus. Download FIG S1, TIF file, 1.1 MB. Copyright © 2021 Junkermeier and Hengge. 2021 Junkermeier and Hengge. https://creativecommons.org/licenses/by/4.0/ This content is distributed under the terms of the Creative Commons Attribution 4.0 International license.

Other exopolysaccharide synthesis systems, e.g., bacterial cellulose synthase or poly-β-1,6-GlcNAc (PGA) synthase, are commonly activated by c-di-GMP. NfrB, however, seems to be a glycosyltransferase, which contains a MshEN domain as a potential c-di-GMP binding domain. Therefore, we purified the soluble MshEN domain of NfrB (NfrB^414-745^) and tested whether it binds c-di-GMP in a radial capillary action of ligand assay (DRaCALA). Specific interaction was indeed observed (Fig. 1C), which was specifically outcompeted by excess unlabeled c-di-GMP, but not by any of the other nucleotides tested (Fig. 1C). To determine the binding affinity, we performed microscale thermophoresis (MST) experiments, which not only confirmed c-di-GMP binding to NfrB^414-745^ but also revealed a *K_d_* of 1.0 ± 0.35 μM (Fig. 1D).

### The *nfrBA-ybcH* operon is postexponentially expressed, temperature controlled, and coregulated with flagella.

**FIG 2:**
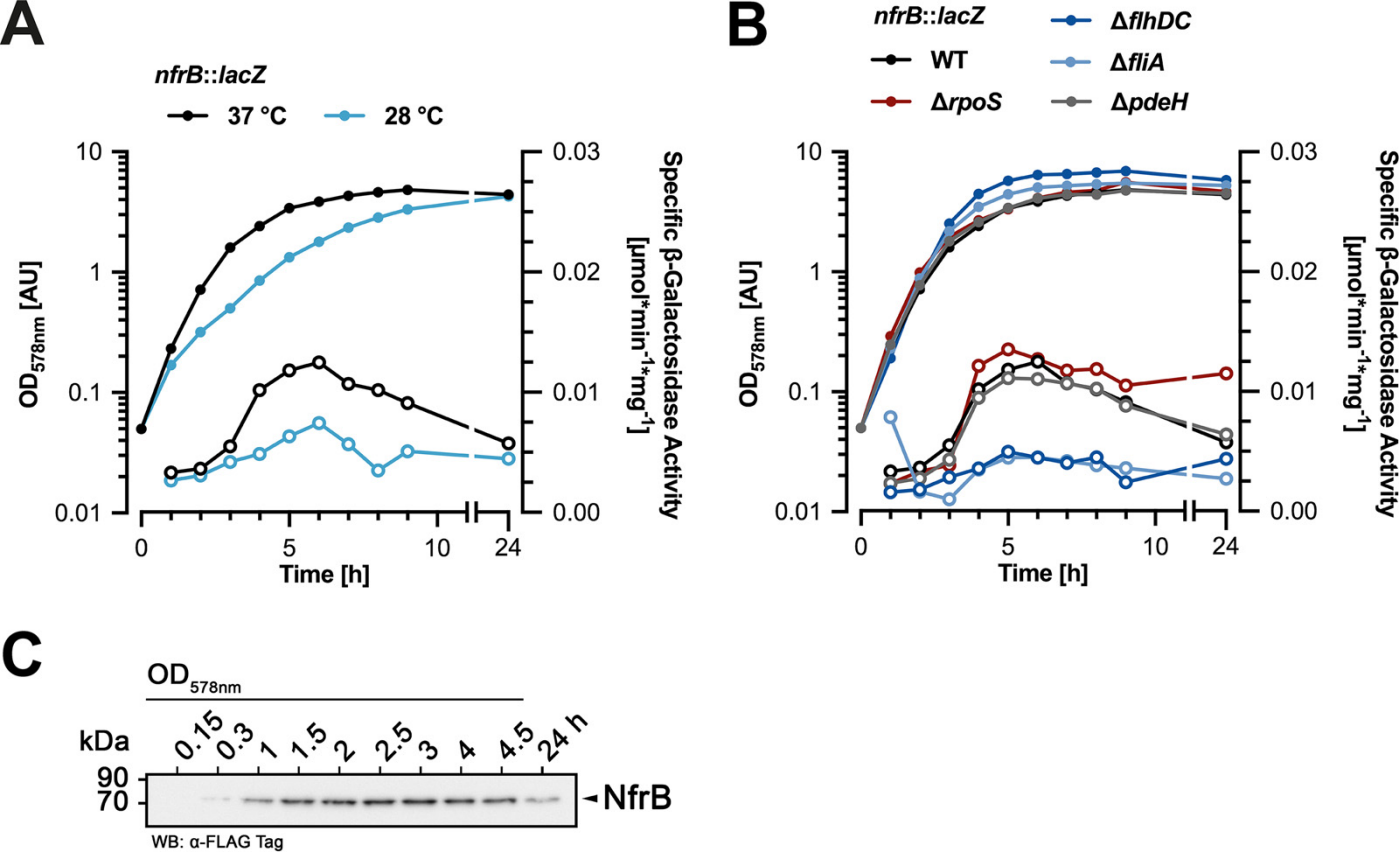
The Nfr system is expressed in postexponentially growing E. coli cells in a temperature-controlled and FliA-activated manner. (A) Expression of the single copy *nfrB*::*lacZ* reporter fusion in the E. coli K-12 strain W3110 during the growth in liquid LB medium. OD_578_ (closed symbols) and specific β-galactosidase activities (open symbols) were determined during growth at 37 or 28°C. (B) Expression of the *nfrB*::*lacZ* reporter fusion in W3110 derivatives carrying additional mutations in *rpoS*, *flhDC*, *fliA*, and *pdeH* was determined as described above for cells growing in liquid LB medium at 37°C. (C) Immunoblot analysis of chromosomally encoded C-terminally 3×FLAG-tagged NfrB in a derivative of strain W3110. Samples were taken at the indicated OD_578_ and after growth for 24 h in liquid LB medium at 37°C.

To gain insights into the physiological function of NfrB in E. coli, we investigated *nfrB* expression and regulation in E. coli. We generated a single-copy *nfrB*::*lacZ* reporter gene fusion, which reflects the promoter activity of *nfrB* (and thus of the entire *nfrBA-ybcH* operon) and monitored its expression during the growth cycle of E. coli in liquid lysogeny broth (LB) medium. Overall, *nfrB*::*lacZ* was expressed at relatively low levels in wild-type cells (Fig. 2A). Expression increased during late exponential phase and again noticeably declined during entry into stationary phase. This pattern was similar, but expression levels were ∼2-fold higher at 37°C than at 28°C (Fig. 2A). The decreasing expression of the *nfrB*::*lacZ* reporter fusion during early stationary phase suggested a negative regulation by the general stress and stationary-phase sigma factor RpoS (σ^S^). Indeed, expression of *nfrB*::*lacZ* remained higher in stationary phase in an *rpoS* mutant background (Fig. 2B).

This transiently increased expression in the late or postexponential phase is a pattern that is typically found for genes that are regulated by the flagellar control cascade, which involves the flagellar master regulator FlhDC and the flagellar sigma factor FliA (σ^28^). Knocking out either FlhDC or FliA indeed reduced the expression of the *nfrB*::*lacZ* reporter gene fusion (Fig. 2B). Yet, the absence of FliA also leads to increased intracellular c-di-GMP level (5), since *pdeH*, which encodes the master PDE in E. coli, is a FliA-dependent flagellar class 3 gene (26). Therefore, we wanted to rule out the possibility that c-di-GMP somehow controls the expression of *nfrB*. However, the loss of *pdeH* had no effect on the expression of *nfrB*::*lacZ* (Fig. 2B). Thus, our data indicate that the *nfrBA-ybcH* operon belongs to the group of flagellar class 3 genes.

In addition, we determined cellular protein levels of NfrB by immunoblot analysis. For this purpose, we inserted a FLAG-tag-encoding sequence close to the 3′ end of *nfrB* in the chromosome. The 3×FLAG-tagged variant of NfrB (NfrB^FLAG^) possesses the tag epitope between A736 and Q737, to avoid any polar effects on the translation initiation of NfrA, since the coding sequences of both genes overlap by 14 bp (Fig. 1B). NfrB^FLAG^ showed increasing abundance during postexponential growth of E. coli, whereas levels declined again during entry into stationary phase (Fig. 2B). In summary, these results show that NfrB is predominantly expressed in E. coli during postexponential growth in a FliA-activated manner.

### Disruption of the *nfr* operon restores the motility defect of a *ΔpdeH ΔycgR* mutant.

**FIG 3:**
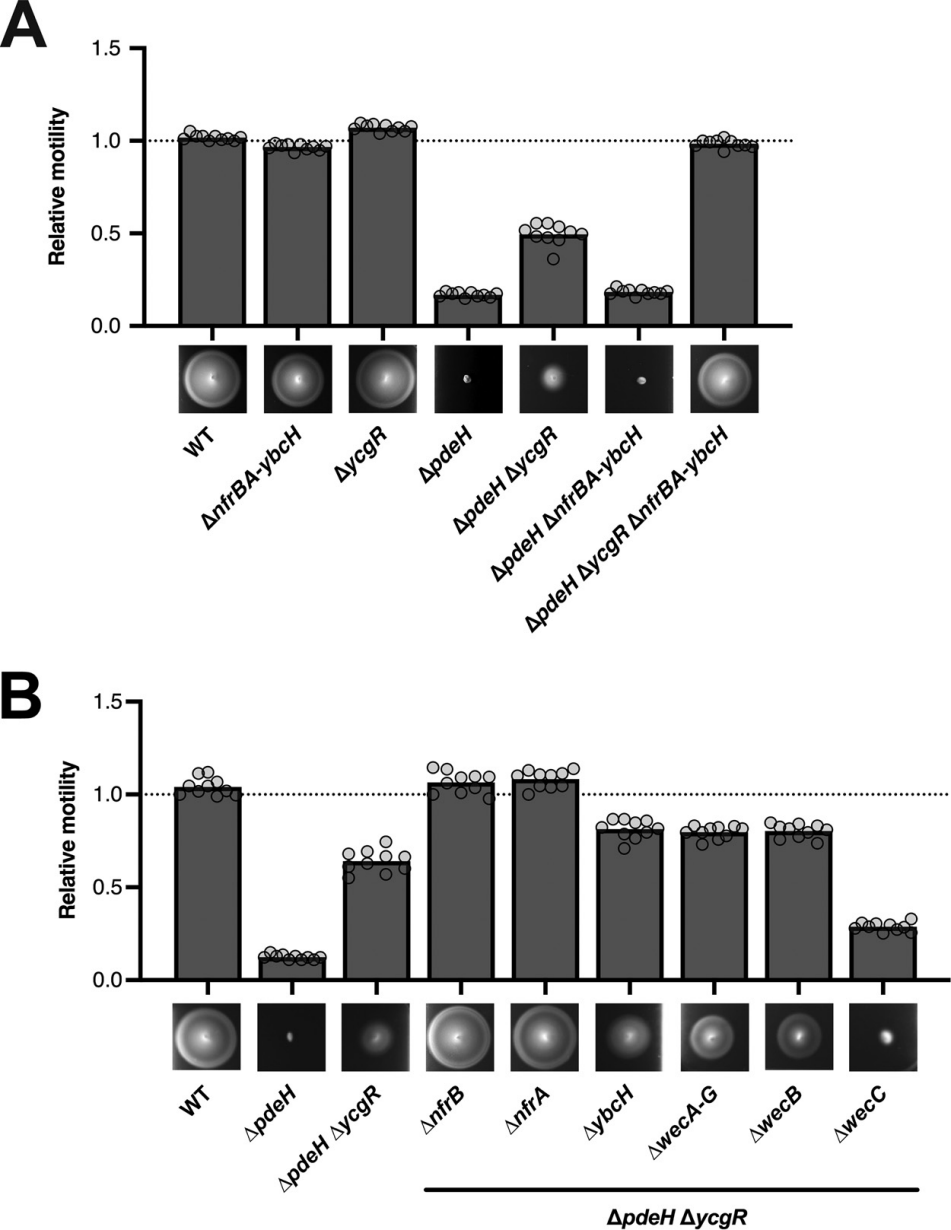
The Nfr system restrains bacterial motility under conditions of elevated intracellular c-di-GMP levels. The motility phenotypes of strain W3110 (WT) and the indicated mutant derivatives were analyzed in TB soft agar plates (0.3% agar) and quantified after 4.5 h of incubation at 37°C. Representative figures are shown at the bottom of the graphs. The diameters of the motility swarm were measured and normalized to the WT strain. The bar graphs represent the means of *n *=* *10 biologically independent samples. Replicates are shown as individual data points (cycles).

The finding that the Nfr system is under the control of the flagellar sigma factor FliA—a property that it shares with some other nonflagellar proteins related to c-di-GMP signaling such as PdeH and the PilZ domain protein YcgR—suggested a physiological and possibly regulatory connection between the Nfr system and bacterial motility. Therefore, we tested swimming motility of a Δ*nfrBA-ybcH* mutant strain in semisolid agar plates but observed no difference compared to the parental strain (Fig. 3A). The loss of the master PDE PdeH renders cells nonmotile (26–28) (Fig. 3A), because the resulting strongly increased intracellular c-di-GMP level (5) activates YcgR, which in its c-di-GMP-bound form functions as a flagellar brake by directly interacting with the flagellar basal body (29, 30). However, knocking out YcgR only partially suppresses this motility defect of a *pdeH* mutant (26), indicating that an additional factor restrains bacterial motility in the *pdeH ycgR* background. This factor seems to be the Nfr system, since deleting also the *nfrBA-ybcH* operon in addition to *pdeH* and *ycgR* restored wild-type motility (Fig. 3A). Similarly, also single gene disruptions of *nfrB* or *nfrA* could restore full motility of a *pdeH ycgR* mutant (Fig. 3B). Interestingly, however, deleting *ybcH* only partially suppressed the *pdeH ycgR* motility defect (Fig. 3B), suggesting a nonessential role for YbcH in the function of the Nfr system. We conclude that under conditions of increased intracellular c-di-GMP levels (probably required to activate NfrB), the Nfr system can interfere with bacterial motility. This motility defect could be explained by the involvement of the Nfr system in synthesis and secretion of a yet-unknown exopolysaccharide.

Based on this hypothesis, we reasoned that also a change in substrate availability for the glycosyltransferase activity of NfrB might affect the motility phenotype. For this reason, we further investigated the role of NfrC (WecB), which is also required for phage N4 infection (22) and which was shown to be an epimerase in the production of UDP-*N*-acetylmannosamine (UDP-ManNAc), a precursor for the enterobacterial common antigen (ECA) (23, 31, 32). Indeed, deletion of either the entire *wec* operon (*wecA-G*, encoding all the enzymes required for the biosynthesis of the ECA) or of *nfrC* (*wecB*) alone also showed a moderate suppression of the *pdeH ycgR* phenotype (Fig. 3B). This finding suggests that NfrB uses the product of NfrC (WecB), i.e., UDP-ManNAc, as a substrate for the synthesis of an exopolysaccharide. Interestingly, disrupting *wecC*—the gene next to *wecB* in the *wecA-G* operon—had the opposite effect, i.e., caused an even greater motility defect of the *pdeH ycgR* mutant background (Fig. 3B). Since ECA synthesis is defective in a *wecC* mutant, more UDP-ManNAc produced by the intact NfrC (WecB) in this strain would be available for the synthesis of the exopolysaccharide produced by NfrB.

### Infection of *E. coli* with bacteriophage N4 requires the activity of the glycosyltransferase domain of NfrB.

**FIG 4:**
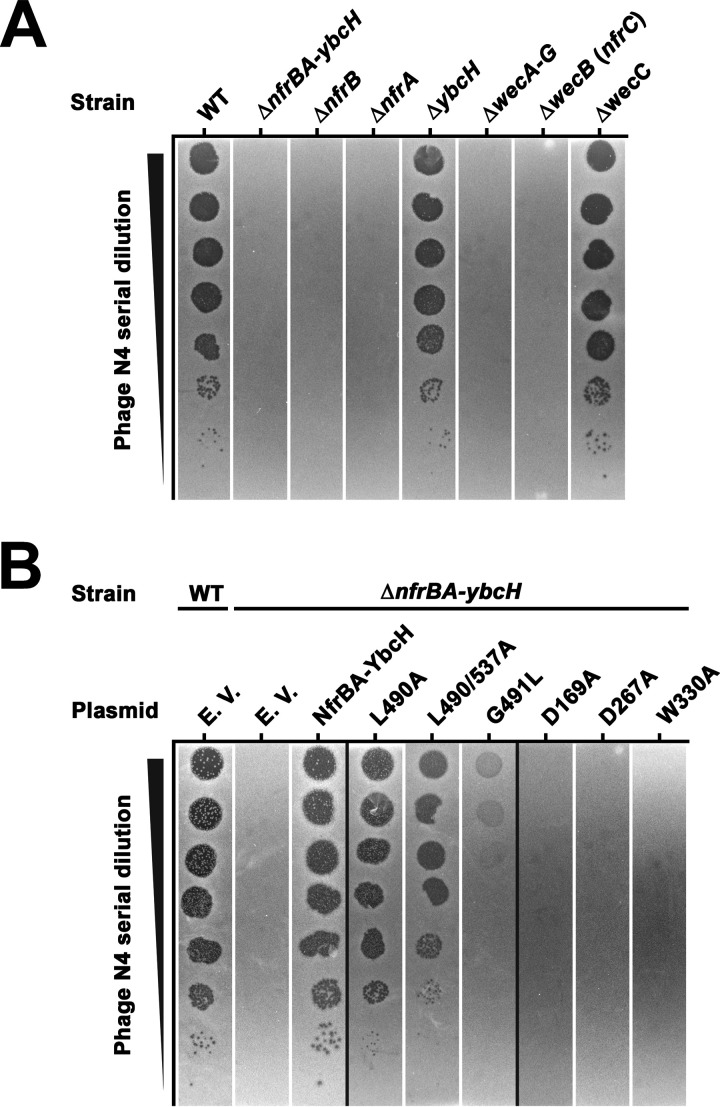
Dissecting the role of the Nfr system in the N4 bacteriophage infection. Plaque formation of phage N4 on E. coli K-12 strain W3110 (WT) was tested using serial dilutions (steps of 1:10 dilutions) of a phage N4 lysate spotted (2 μl) on top-agar (LB medium, 1.1% agar) containing the respective bacterial strains and incubated at 37°C. (A) Plaque formation on strain W3110 and derivatives carrying knockout mutations in the indicated genes. (B) Plaque formation on W3110 (WT) and a derivative strain carrying a deletion removing the entire *nfrBA-ybcH* operon, both transformed with plasmids encoding the wild-type Nfr system (NfrBA-YbcH) or derivatives with the indicated mutations in the MshEN domain (L490A, L490/537A, and G491L) or the glycosyltransferase domain (D169A, D267A, and W330A) of NfrB. The empty vector (EV) was used as a control.

NfrA, NfrB, and NfrC (WecB) have been implicated in the infection of E. coli with phage N4 (19, 22). Based on the results described above, we were inspired to gain further insights into the molecular function of the Nfr system by revisiting its role in phage N4 infection. We first analyzed the ability of phage N4 to lyse different derivatives of E. coli K-12 W3110 using spot assays. As expected, phage N4 was not able to lyse E. coli mutant devoid of NfrB or NfrA, whereas YbcH was dispensable for successful phage infection (Fig. 4A). In addition, a knockout of the biosynthetic pathway of ECA (a deletion of the entire *wec* operon), as well as a single gene disruption of *nfrC* (*wecB*) showed a phage N4 plating defect. Remarkably, knocking out WecC alone, which also results in the absence of ECA, did not change the efficiency of plating of phage N4, indicating that the only contribution of the ECA system to phage N4 infection is the UDP-ManNAc produced by NfrC (WecB). This in turn suggests that the exopolysaccharide produced by NfrB—and not only the NfrA and NfrB proteins *per se*—are involved in phage infection.

In order to further dissect the roles of the glycosyltransferase (GT) und MshEN domains of NfrB in phage N4 infection, the *nfrBA-ybcH* operon was cloned onto a low-copy-number plasmid vector (pAP58), with the *tac* promoter (p_tac_) driving its expression. No inducer (IPTG [isopropyl-β-d-thiogalactopyranoside]) was added to the media in order to obtain just moderate (leaky) expression from p_tac_ in our experiments, since the *nfr* operon is expressed at low levels from the chromosome. In addition, we introduced mutations in highly conserved residues of NfrB that are crucial (i) for the glycosyltransferase activity (D169A, D267A, and W330A) (25) and (ii) for c-di-GMP binding in the MshEN domain (L490A, L490/537A, and G491L) (17). None of these mutations affected the cellular levels of NfrB (see Fig. S2). When introduced into a Δ*nfrBA-ybcH* mutant strain, the wild-type construct restored the plating efficiency of phage N4 (Fig. 4B). However, the variants lacking the active-site amino acids of the glycosyltransferase domain of NfrB failed to complement the phage N4 plating defect (Fig. 4B). Variants with both single (L490A) and double (L490/537A) amino acid substitutions in the MshEN domain showed a moderate but additive reduction in the N4 plaque forming efficiency, whereas the mutation of G491 in the MshEN domain led to the most profound reduction in the N4 plating efficiency (Fig. 4B). Thus, more than one such change seems to be needed to prevent c-di-GMP binding completely, which is in line with previous similar results with MshE from V. cholerae (17). Together, these results show that phage N4 does not only use NfrB and NfrA proteins as the host receptors for infection but requires both the glycosyltransferase activity of NfrB and its ability to bind c-di-GMP. This indicates that binding of c-di-GMP to the MshEN domain of NfrB allosterically activates its GT domain. This conclusion is in line with the finding reported above that a mutation specifically in *nfrC* (*wecB*), which eliminates the synthesis of the putative substrate of the GT domain, i.e., UDP-ManNAc, confers resistance to phage N4 infection.

10.1128/mbio.03249-21.2 FIG S2 Single amino acid substitution at relevant positions do not affect cellular levels of NfrB. NfrB^FLAG^A-YbcH (with the wild-type sequence, as well as with the indicated amino acid substitutions) was expressed from plasmid pAP58 in an E. coli K-12 strain W3110 derivative strain carrying a deletion of the *nfrBA-ybcH* operon. Samples were taken from cells grown in liquid LB medium at 37°C for 6 h. The cellular levels of NfrB^FLAG^ were analyzed by immunoblot analysis. Download FIG S2, TIF file, 0.8 MB. Copyright © 2021 Junkermeier and Hengge. 2021 Junkermeier and Hengge. https://creativecommons.org/licenses/by/4.0/ This content is distributed under the terms of the Creative Commons Attribution 4.0 International license.

### Specifically DgcJ is required for NfrBA-dependent phage N4 infection and directly interacts with NfrB.

**FIG 5:**
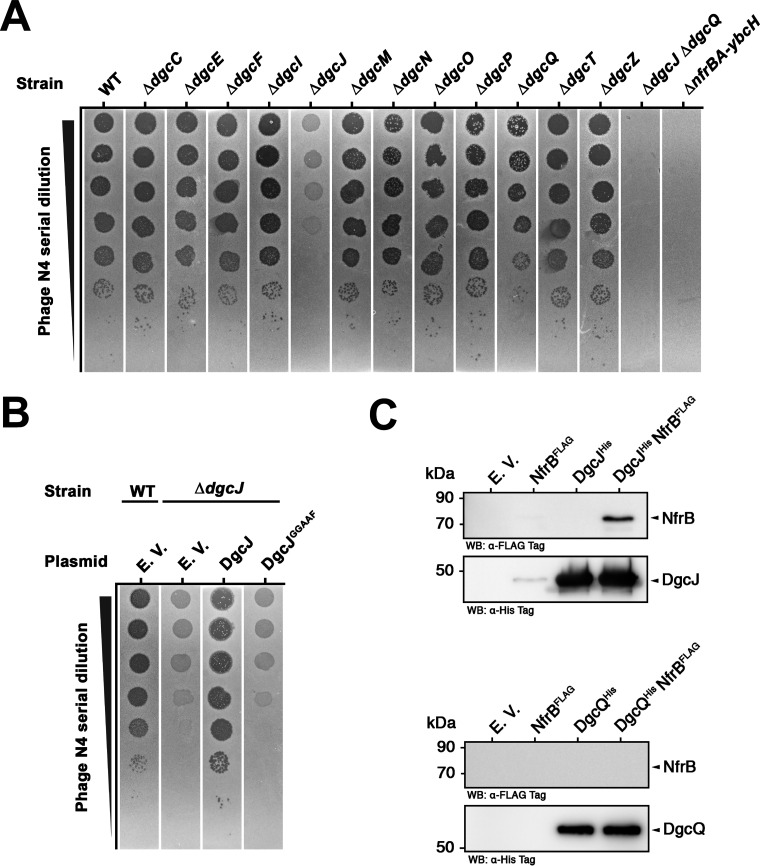
DgcJ is required for phage N4 infection and directly interacts with NfrB. (A) Plaque formation of phage N4 on strain W3110 (WT) and derivates carrying single deletion mutations in all 12 genes encoding active DGCs, a double deletion of *dgcJ* and *dgcQ* or a deletion of the entire *nfrBA-ybcH* operon was assayed as described in Fig. 4. (B) Plaque formation on a W3110 derivative carrying a deletion in *dgcJ* transformed with a plasmid encoding DgcJ or DgcJ with an active-site mutation in its GGDEF domain (DgcJ^GGAAF^). The empty vector (EV) was transformed as a control. (C) NfrB copurifies with DgcJ. DgcJ^His^ was expressed from the medium copy number vector pRH800 in the presence of NfrB^FLAG^, expressed from the low-copy-number plasmid pAP58. Affinity chromatography was performed with the indicated cellular extracts on Ni-NTA resin, which specifically binds the 6×His epitope of DgcJ^His^. In a similar parallel approach, DgcQ^His^ was purified in the presence of NfrB^FLAG^. Empty vectors were used as controls in combinations as indicated. Eluates were analyzed on SDS polyacrylamide gels, followed by visualization of NfrB (87 kDa), DgcJ (56 kDa) and DgcQ (65 kDa) by immunoblotting with anti-Flag (upper panels) and anti-His_6_ antibodies (lower panels), respectively.

One criterion to identify local c-di-GMP signaling is the observation that knocking out distinct DGCs or PDEs leads to highly specific phenotypes (7). Our finding that the loss of the ability of NfrB to bind c-di-GMP conferred resistance against the infection with phage N4 (Fig. 4B), raised the question, whether a specific DGC could be required for successful phage infection. Therefore, all the single knockout mutants lacking the 12 active DGCs of E. coli K-12 were screened for a phage N4 plating defect (Fig. 5A). In fact, a *dgcJ* deletion showed a severe plating defect, while a *dgcQ* deletion marginally reduced plating efficiency by 1 order of magnitude. Notably, the *dgcJ dgcQ* double-knockout mutant did not show any detectable plaque formation of phage N4 (Fig. 5A). Thus, NfrB seems specifically activated by DgcJ, with DgcQ providing for a minor backup.

Next, we focused on the role of DgcJ in phage N4 infection. Therefore, the *dgcJ* gene was cloned on a medium copy number plasmid (pRH800), with p_tac_ driving its expression. No inducer (IPTG) was added to the media in order to not drastically overproduce DgcJ. In addition, we constructed a derivative with active site (A-site) mutations (DgcJ^GGAAF^) to eliminate the DGC activity of the GGDEF domain of DgcJ. When introduced into the *dgcJ* deletion mutant, wild-type DgcJ was able to complement the phage N4 plating defect, whereas DgcJ^GGAAF^ failed to do so (Fig. 5B). These data show that the N4 infection does not simply require the presence of the inner membrane protein DgcJ but, more specifically, the production of c-di-GMP by DgcJ. Therefore, DgcJ is involved in a highly specific c-di-GMP-mediated activation of NfrB.

Specific signaling of a distinct DGC (or PDE) to a particular effector/target system can be expected to occur via a direct protein-protein interaction (7). To test for such interaction, we added a C-terminal 6×His tag to DgcJ (DgcJ^His^) expressed from pRH800. In parallel, a similar construct on the same vector was obtained with DgcQ (DgcQ^His^), which had shown a minor backup activation of NfrB (Fig. 5A). The coding sequence for NfrB^FLAG^ was cloned together with *nfrA* and *ybcH* on the compatible low-copy-number vector pAP58, which allows cotransformation and coexpression of NfrB^FLAG^ and DgcJ^His^ (or DgcQ^His^). All of these proteins were expressed and, when coexpressed, did not affect each other’s level of expression (see Fig. S3). This allowed affinity chromatography or “pulldown” experiments, where DgcJ^His^ or DgcQ^His^ are bound and eluted from a nickel-charged affinity (Ni-NTA) resin (Fig. 3C). When NfrB^FLAG^ was coexpressed with DgcJ^His^, it indeed coeluted with DgcJ^His^ (Fig. 3C). This NfrB^FLAG^/DgcJ^His^ interaction was highly specific, since NfrB^FLAG^ alone was not retained by the Ni-NTA resin and did not copurify with DgcQ^His^ (Fig. 3C).

10.1128/mbio.03249-21.3 FIG S3 Relevant protein levels in cellular samples used for the copurification experiment shown in Fig. 5. Protein levels were analyzed by immunoblot analysis for DgcJ^His^ (A), NfrB^FLAG^ (B and D), and DgcQ^His^ (C) before the cell lysis procedure was applied. Download FIG S3, TIF file, 1.5 MB. Copyright © 2021 Junkermeier and Hengge. 2021 Junkermeier and Hengge. https://creativecommons.org/licenses/by/4.0/ This content is distributed under the terms of the Creative Commons Attribution 4.0 International license.

In conclusion, among all DGCs of E. coli, it is specifically DgcJ that is required for NfrB-dependent infection with phage N4. This role of DgcJ involves its ability to synthesize c-di-GMP. The activation of NfrB by this locally produced c-di-GMP is supported by the direct and specific protein-protein interaction between NfrB and DgcJ.

### NfrB is locally and specifically activated by DgcJ even though DgcQ and DgcE are active in parallel.

In principle, our finding that the activation of NfrB depends specifically on the diguanylate cyclase activity of DgcJ would also be compatible with the possibility that DgcJ may be the only active DGC under the conditions tested (i.e., that the contribution of other DGCs to the global intracellular concentration of c-di-GMP might be negligible). Hence, we addressed the question of whether other DGCs are active during vegetative growth at 37°C and thus drive up c-di-GMP levels in the *pdeH* mutant, which—via YcgR—interferes with motility.

**FIG 6:**
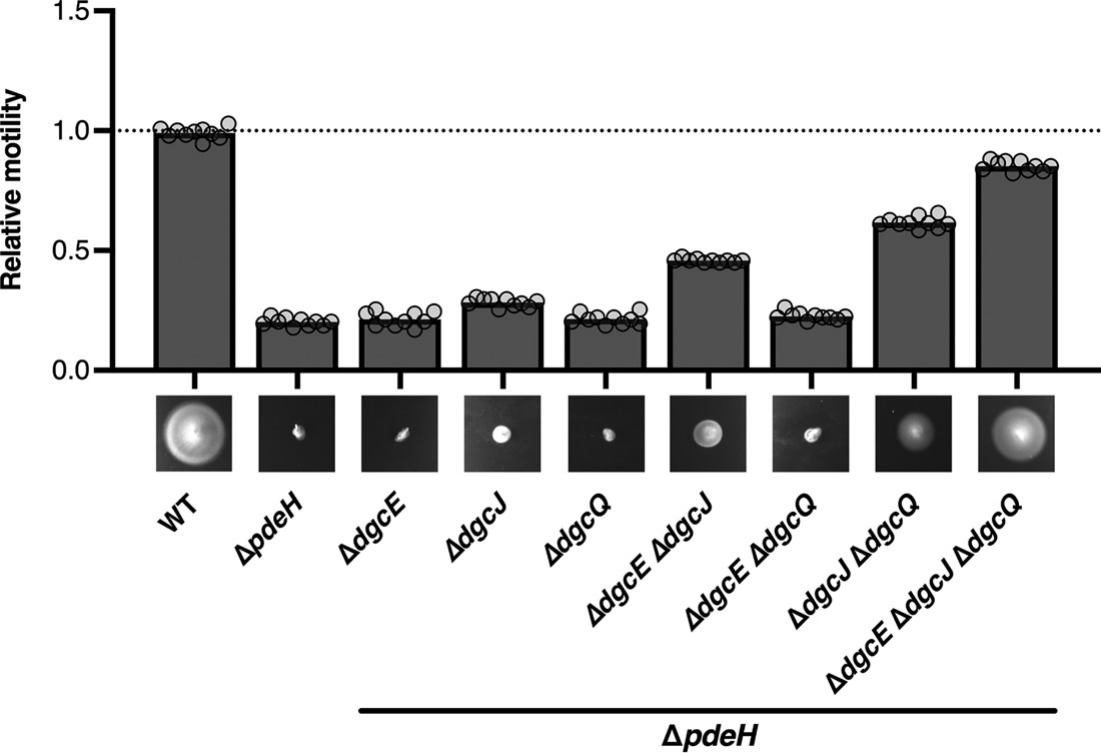
DgcJ, DgcQ, and DgcE all contribute to inhibiting motility of a *pdeH* mutant. Motility phenotypes of strain W3110 (WT) and derivatives carrying the indicated mutations in *pdeH*, *dgcE*, *dgcJ*, and *dgcQ* were analyzed in TB soft agar plates (0.3% agar) and quantified after 4.5 h of incubation at 37°C. Representative figures are shown at the bottom of the graphs. The diameters of the motility swarms were measured and normalized to the WT strain. The bar graphs represent the mean of *n *=* *10 biologically independent samples. Replicates are shown as individual data points (cycles).

To test this, we examined whether eliminating other DGCs could suppress the *pdeH* motility defect. When knocked out alone, only the *dgcJ* deletion could relieve the motility defect of the *pdeH* mutant to some extent, whereas deleting either *dgcQ* or *dgcE* had no effect (Fig. 6). However, when, in addition to *dgcJ*, *dgcQ* or *dgcE* was also knocked out, additive effects were observed. Eliminating DgcJ, DgcQ, and DgcE all together fully restored the motility of the *pdeH* mutant (Fig. 6). These results indicate that in vegetatively growing cells at 37°C, DgcJ, DgcQ, and DgcE are all active and contribute to a global pool of c-di-GMP, which in the absence of the master PDE PdeH becomes high enough to inhibit motility via YcgR. However, under conditions where PdeH is present to constantly drain the cellular c-di-GMP pool (5), which allows for motility as YcgR is not activated, it is only DgcJ that can specifically activate NfrB by direct interaction (Fig. 5C) and thereby allow phage N4 infection.

### Phylogenetic analysis of the NfrBA-YbcH system shows its frequent genetic linkage to NfrC (WecB)-like enzymes and DGCs.

**FIG 7:**
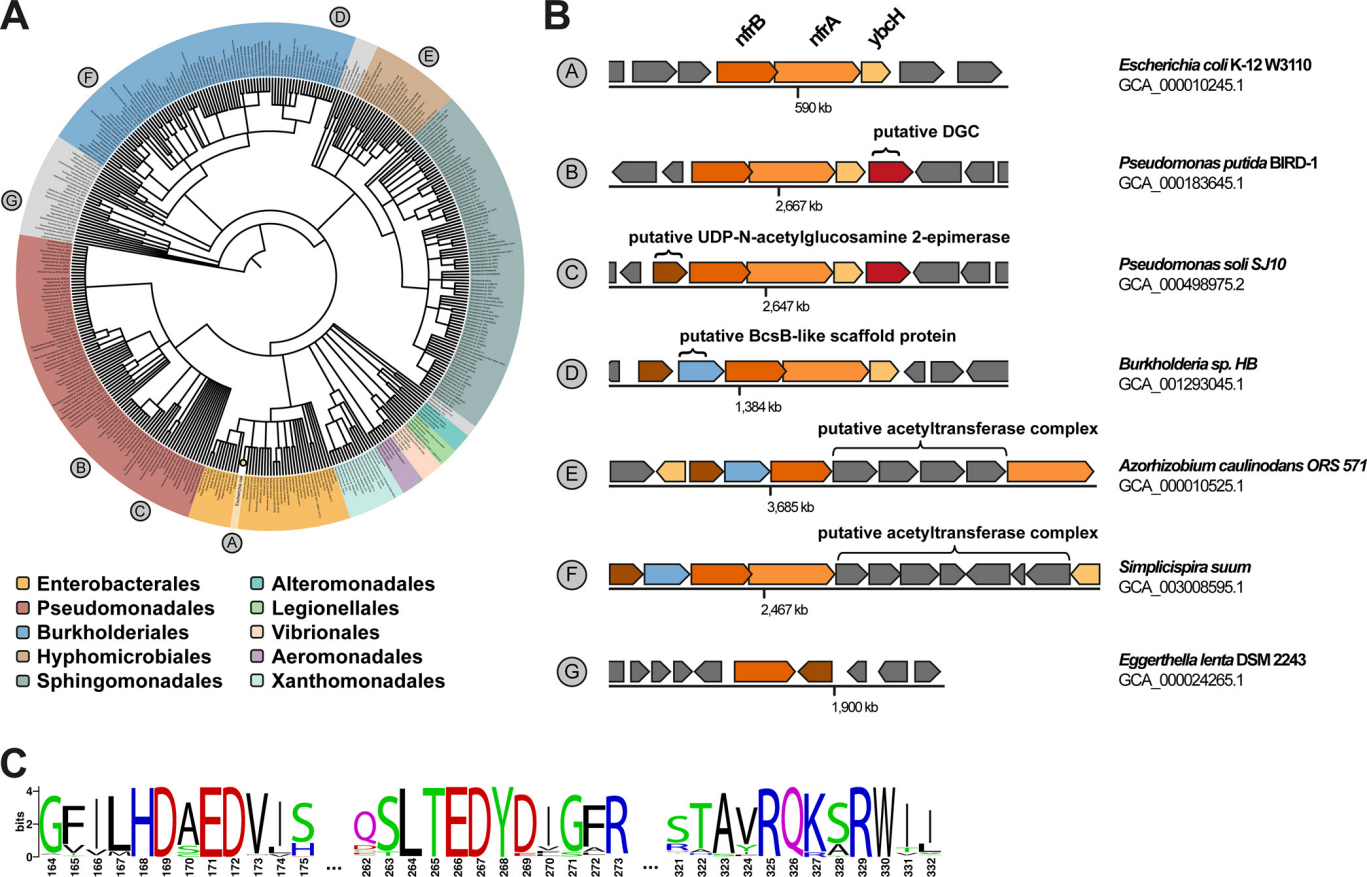
Genomic context and NfrB homologs in various bacterial species. (A) A phylogenetic tree based on the NCBI taxonomy of 406 bacterial species (beyond E. coli) encoding NfrB homologes was generated by phyloT (http://itol.embl.de/) and visualized with iTOL (v6.3.2). Bacterial orders are highlighted by the indicated colors. The E. coli node is collapsed (single yellow circle). (B) Schematic representations of operons encoding NfrB homologs and their respective genetic background of representative strains, whose position in the phylogenetic tree is shown in panel A. (C) Sequence logo of the 1,842 NfrB homologs identified in this study showing the highly conserved active-site features of the glycosyltransferase domains. The numbering is according to the corresponding amino acids in NfrB of E. coli (see Fig. 1A).

Finally, we analyzed the phylogenetic distribution of NfrB homologs in various bacterial clades with a special focus on the genomic neighborhoods of the respective genes using TBLASTN searches. A total of 1,841 NfrB (*Ec*NfrB) homologs were found, 1,101 (60%) of which were encoded in different E. coli strains, while the remaining ones were present in 406 taxonomically different bacterial species (Fig. 7A). *Ec*NfrB homologs can be found predominantly in gamma- and betaproteobacteria but, occasionally, also occur in alphaproteobacteria and the delta and epsilon subdivisions of proteobacteria. All of the identified homologs showed the conserved DxD, TED, and QxxRW active-site motif in the GT domain (Fig. 7C), and a large majority of 1,705 homologs also featured a conserved MshEN domain.

Strikingly, in all organisms identified, which do not encode at least one NfrC (WecB) homolog (i.e., the UDP-*N*-acetylglucosamine 2-epimerase) in a different biosynthetic pathway (such as the ECA pathway in E. coli), a gene encoding a NfrC (WecB) homolog can be found directly associated with the respective NfrB coding sequence (for example, in Pseudomonas soli SJ10 [GCA_000498975.2]) (Fig. 7B). Moreover, in most pseudomonads, a GGDEF domain protein, i.e., a putative DGC, can be found associated with the *nfr* operon (Fig. 7B). In some cases, e.g., in *Simplicispira suum* (GCA_003008595.1), we identified an additional cluster of genes integrated into the *nfr* gene cluster, which seems related to the acetyltransferase complex that modifies the exopolysaccharide alginate in Pseudomonas aeruginosa (33). In rare cases, NfrB homologs can also be found in Gram-positive bacteria such as Eggerthella lenta DSM 2243 (GCA_000024265.1), which evidently lacks the outer membrane pore (NfrA).

To summarize, our analysis of the local genomic associations of genes encoding NfrB homologs provides further evidence that the system uses UDP-ManNAc as a substrate to produce an exopolysaccharide. In some cases, this exopolysaccharide may be even modified by an acetyltransferase machinery. The presence of a putative DGC gene immediately downstream, i.e., potentially as a fourth gene in a full *nfrBA-ybcH* operon in some Pseudomonas spp. suggests a specific role of the respective DGC for the Nfr system in these bacteria.

## DISCUSSION

### NfrB is a novel c-di-GMP-binding effector component locally controlled by DgcJ.

The highly conserved MshEN domain of NfrB (Fig. 1A) was a strong indication that NfrB represents a novel c-di-GMP binding effector in E. coli. NfrB indeed binds c-GMP specifically (Fig. 1C) with a *K_d_* of 1 ± 0.35 μM (Fig. 1D). Thus, NfrB has an affinity in the same range as that of other c-di-GMP effectors in E. coli, as exemplified by a *K_d_* values of 0.84 μM for YcgR (27) or 8.2 μM for BcsA (34). Due to the activity of the strongly expressed “master” PDE PdeH, E. coli maintains remarkably low intracellular c-di-GMP levels, ranging from as low as 40 to 50 nM in vegetatively growing cells (an optical density at 578 nm [OD_578_] = 1) to approximately 80 to 100 nM during the transition into stationary phase (OD_578_ = 3) (5). With a *K_d_* that is at least 10-fold higher, NfrB should thus mainly be in the c-di-GMP-free (hence inactive) state under these conditions—if it responds just to the global intracellular c-di-GMP concentration. However, the finding that NfrB is active under these conditions, as demonstrated by the ability of phage N4 to infect E. coli in an Nfr-dependent manner (Fig. 4), suggests local activation by c-di-GMP.

**FIG 8:**
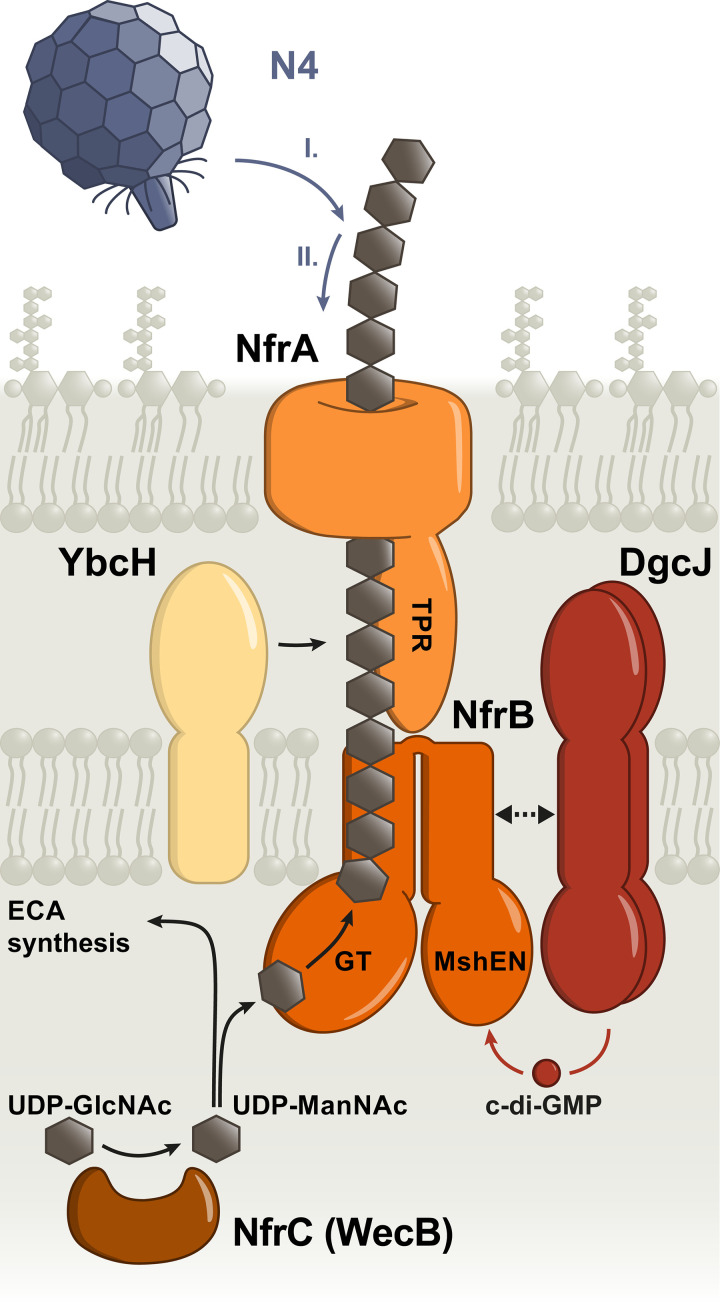
Model of the Nfr/DgcJ system and its role in locally c-di-GMP-activated exopolysaccharide production and bacteriophage N4 adsorption. DgcJ and NfrB colocalize via a direct protein-protein interaction. The C-terminal MshEN domain of NfrB binds c-di-GMP specifically produced by DgcJ, leading to an allosteric activation of the N-terminal glycosyltransferase domain of NfrB. WecB converts UDP-GlcNAc into UDP-ManNAc, which is used for the biosynthesis of the ECA. In addition, the glycosyltransferase domain of NfrB uses UDP-ManNAc as a substrate to produce a putative ManNAc-polymer, which is secreted by the outer membrane protein NfrA. YbcH is a periplasmatic protein, which may play an auxiliary role, but is not essential for polysaccharide secretion. Phage N4 binds the exopolysaccharide secreted by the Nfr system as an initial receptor (I) before interacting with NfrA (II), which leads to the irreversible adsorption of the phage.

Such an active output of a c-di-GMP-controlled process at global cellular c-di-GMP levels severalfold below the *K_d_* of the relevant c-di-GMP-binding effector is one of several criteria that should all be met to unequivocally establish a case of local c-di-GMP signaling (7). Another criterion is the direct interaction between the specific DGG (and/or PDE) and effector/target component(s) in a signaling protein complex. Such physical vicinity increases the probability of c-di-GMP produced by the specific DGC to either hit the effector binding site or that of a colocalized PDE (7, 11). DgcJ and NfrB were indeed found to directly interact (Fig. 5C). The inability of an A-site point mutation in DgcJ (DgcJ^GGAAF^) to complement the Δ*dgcJ* phenotype (Fig. 5B) indicates that DgcJ functions to provide c-di-GMP locally, so it has a high chance of hitting the MshEN domain of NfrB, resulting in the activation of its GT domain (Fig. 8). In other words, the direct interaction between DgcJ and NfrB plays a scaffolding role similar to DgcC serving BcsA (11), i.e., serves to establish close proximity rather than also having a direct regulatory impact.

The third criterion for local c-di-GMP signaling are specific phenotypes of mutations that eliminate particular DGCs or PDEs, but not of mutations in other DGCs or PDEs that are concomitantly expressed and active (7). This criterion is also met by the DgcJ/NfrB system, since the ability of phage N4 to infect E. coli in a NfrB-mediated manner strongly and specifically depends on the catalytic activity of DgcJ (Fig. 5A and B), even though DgcQ and DgcE are active under the same conditions (in cells growing at 37°C), as evidenced by the additive effects of all these three DGCs on motility in the absence of the master PDE PdeH (Fig. 6). These observations also confirm the previous insight that for c-di-GMP signaling to act locally, a high active and/or abundant master PDE like PdeH is required to maintain a very low global cellular c-di-GMP pool (5). Notably, the *dgcQ* mutation still had a slight effect on the plating efficiency of phage N4 (Fig. 4A). However, DgcQ did not coelute with NfrB in our experiments (Fig. 5C), indicating that DgcQ is not as specifically involved in the control of NfrB as DgcJ. However, due to the common membrane location of DgcQ and NfrB, DgcQ-produced c-di-GMP may still have some probability to reach NfrB before it is eliminated by PdeH. Based on all these criteria for local c-di-GMP signaling being met here, we therefore propose that DgcJ provides a local source of c-di-GMP right next to NfrB and thereby activates the Nfr system specifically even in the presence of additional active DGCs.

In parallel to our work, another study also reported that c-di-GMP is required for the infection with phage N4 (35). In good agreement with our data, it was found that a *dgcJ* mutant showed stronger fitness in the presence of phage N4, whereas the overexpression of six PDEs (PdeO, PdeR, PdeN, PdeL, PdeB, and PdeI) resulted in a resistance phenotype. That only 6 of the 13 PDEs of E. coli K-12 were able to confer a resistance phenotype may be due to the remaining PDEs being catalytically inactive under the conditions tested or just not present in the Dub-seq library used (35). However, the finding that an ectopic expression of various PDEs increases resistance against phage N4 is in line with our proposed local signaling model for NfrB, since NfrB and DgcJ form a specific, yet open signaling module. This allows the DgcJ-produced c-di-GMP to either bind to the MshEN domain of NfrB or diffuse into the cytoplasm, where it gets degraded by PdeH. Expressing additional PDEs from a high-copy-number plasmid (as in reference 35) strengthens the global sink for c-di-GMP in these cells, which will shift the binding equilibrium of NfrB to the c-di-GMP-free and therefore inactive state, which results in the reported phage N4 insensitivity.

Finally, the accompanying publication by Sellner et al. (24) shows that, in particular, PdeL overexpression confers complete phage N4 resistance. With its LuxR-EAL domain architecture, this “trigger PDE” (13) acts a DNA-binding repressor, which downregulates its own gene in a manner that is allosterically controlled by its c-di-GMP binding and PDE activity (36). Thereby, PdeL does not only reduce the cellular c-di-GMP level in a positive-feedback loop, but the new work shows that it also targets additional genes, including the *wec* operon, thereby preventing the expression of NfrC (WecB) (24). Thus, c-di-GMP plays a dual regulatory role in the production of the NfrB-synthesized polysaccharide by controlling *wec* operon transcription and thus the production of the precursor UDP-ManNAc, as well as the glycosyltransferase activity of NfrB.

### The NfrBA system is postexponentially induced, temperature controlled, and coregulated with flagella and can impede motility.

The Nfr system is induced during the posttranscriptional phase of the growth cycle and is transcriptionally regulated by the flagellar control cascade consisting of FlhDC and the sigma factor FliA (Fig. 2B). In addition, it is more strongly expressed at 37°C than at 28°C (Fig. 2A). A similar temperature regulation has also been described for *dgcJ* (previously termed *yeaJ*) (4), which directly stimulates NfrB activity (see above). Overall, this regulatory pattern for the synthesis of the Nfr system and its exopolysaccharide product suggests a physiological function for the NfrB-synthesized exopolysaccharide that may be important within the human host, e.g., in providing protection against recognition by the immune system. Interestingly, lasting upregulation upon upshift to 37°C was recently observed also for other flagellar genes (37), suggesting that temperature input into the Nfr system is connected to its transcriptional regulation by the FlhDC-FliA cascade.

The Nfr systems shares its coregulation with flagella with several other factors that are involved in c-di-GMP signaling in E. coli, i.e., the master PDE PdeH, the c-di-GMP-binding effector YcgR (26) and the GTPase system RdcA/RdcB that eventually—upon a decrease in cellular GTP—directly activates DgcE (38). The coregulation with PdeH is vital for a precise DgcJ-specific control of NfrB activity, since local c-di-GMP signaling depends on a low global c-di-GMP level being maintained by PdeH. On the other hand, YcgR, RdcA/RdcB, and the Nfr system all share the ability to tune down motility in a c-di-GMP-controlled manner, albeit through different mechanisms. RdcA/RdcB-activated DgcE provides c-di-GMP that allows YcgR to operate as a c-di-GMP-activated brake, which binds to the flagellar basal body and thereby directly inhibits flagellar rotation from inside the cell (26, 28–30). In contrast, NfrB is a c-di-GMP-activated glycosyltransferase (Fig. 1) which produces an exopolysaccharide that gets secreted via the outer membrane β-barrel protein NfrA (Fig. 8). Along with YcgR, the Nfr system (and therefore most likely its still uncharacterized exopolysaccharide) reduces motility in a *pdeH* mutant (Fig. 3). Interestingly, this role of the Nfr system seems analogous to the ability of the exopolysaccharide cellulose to restrain motility of a *pdeH ycgR* mutant of Salmonella (39). Our BLAST searches revealed that Salmonella does not possess a NfrB homolog, and the E. coli K-12 W3110 strain used in our motility assays does not produce cellulose (40). Thus, the Nfr-synthesized exopolysaccharide could restrain flagellar rotations by means of steric hindrance in a manner similar to that suggested for cellulose for Salmonella (39).

### What kind of polysaccharide does NfrB produce?

The observed effect on motility also correlated with the availability of cellular UDP-ManNAc, since a knockout mutation of *nfrC* (*wecB*) suppressed the motility defect, while it was enhanced by deleting *wecC* (Fig. 3B). Both gene products are enzymes involved in the production of the ECA. NfrC (WecB) is a UDP-*N*-acetylglucosamine 2-epimerase responsible for the reversible epimerization between UDP-*N*-acetylglucosamine (UDP-GlcNAc) and UDP-*N*-acetylmannosamine (UDP-ManNAc). WecC catalyzes the following step in the biosynthesis of ECA, in which UDP-N-acetyl-mannosaminuronic acid (UDP-ManNAcUA) is synthesized by a dehydrogenation of UDP-ManNAc. Thus, a *nfrC* (*wecB*) mutant lacks UDP-ManNAc, whereas a *wecC* mutant should have higher levels of UDP-ManNAc, since the next step in ECA synthesis is blocked. Based on the observation that (i) *nfrC* (*wecB*) and *wecC* mutations have opposite effects on the motility phenotype (Fig. 3C) and (ii) NfrC is required for phage N4 infection (Fig. 4A) (22), we propose that the GT domain of NfrB uses UDP-ManNAc as a substrate for the polymerization of a polysaccharide. In wild-type cells, NfrB has to compete with WecC for UDP-ManNAc. In a *wecC* mutant, however, higher UDP-ManNAc levels probably increase the rate of production and secretion of the NfrB-synthesized polysaccharide and thereby steric hindrance of flagellar rotation. That NfrB uses UDP-ManNAc as a substrate is also supported by our finding that every bacterial species that we found to possess a NfrB homolog, but no NfrC (WecB) homolog associated with a separate biosynthetic pathway (such as the ECA pathway), shows direct genomic association of its respective *nfrB* and *nfrC* (*wecB*) coding sequences (Fig. 7).

### The NfrBA-produced polysaccharide serves as the primary receptor for phage N4.

The adsorption of tailed phages to their Gram-negative host surface is a stepwise process. It often includes an initial reversible binding of the phage to cell envelope structures, such as surface-exposed glycans or glycosylated structures. As a secondary step, host receptors in the outer membrane are irreversibly bound, which triggers tail contraction and ejection of the phage DNA into the bacterial cell (reviewed in reference 41). Theoretically, phage N4 could bind to the ECA as its initial host receptor. However, in various phage N4 infection studies (20, 35) only mutations in *nfrC* (*wecB*) and none in the other genes of the ECA synthetic gene cluster were found, which argues against a role of ECA in phage N4 infection. Our data indicate that the Nfr system most likely uses UDP-ManNAc—the enzymatic product of NfrC (WecB)—as a substrate for the production of an exopolysaccharide. Importantly, mutations in the glycosyltransferase domain of NfrB, as well as its c-di-GMP-binding MshEN domain, were also found to generate a phage resistance phenotype (Fig. 4B). We therefore propose that phage N4 uses the NfrB-synthesized exopolysaccharide as its initial receptor (Fig. 8). Based on a similar conclusion, the accompanying study proposes NGR (N4 Glycan Receptor) as a name for this exopolysaccharide (24). Moreover, quite a low abundance of the Nfr system of three to five copies per cell was reported (20). Consequently, an initial binding to a secreted polysaccharide and subsequent directional movement may be the most efficient way for phage N4 to reach its final host receptor NfrA. Its role as an initial phage receptor also suggests that the exopolysaccharide is not shed from the cells, which could turn it into a dead-end trap for the phages, but remains surface associated.

## MATERIALS AND METHODS

### Bacterial strains and growth conditions.

The strains used in this study are derivatives of E. coli K-12 strain W3110 (42). C-terminally 3×FLAG-tagged chromosomally encoded constructs of NfrB (NfrB^FLAG^) were generated by a two-step method similar to the one-step-inactivation protocol (43), as described previously (44), using the oligonucleotides listed in Table S1. Knockout mutations in *nfrBA-ybcH*, *nfrB*, *nfrA*, *ybcH*, *wecB*, *wecC*, and the entire *wecA-wzzE-wecBC-rffGH-wecDE-wzxE-wecF-wzyE-wecG* operon are full open reading frame (*orf*) deletions or antibiotic resistance cassette insertions generated by one-step inactivation (43) using the oligonucleotides listed in Table S1. The mutations in all GGDEF/EAL domain-encoding genes as well as in *ycgR*, *pdeH*, *flhDC*, *fliA*, and *rpoS* are full *orf* deletion/resistance cassette insertions generated in W3110 and were previously described (4, 5, 28, 45). When required, cassettes were removed as described previously (43). P1 transduction (46) was used to transfer the mutations. E. coli strain BL21 Gold (Stratagene catalog number 230130) (47) was used for the protein purification experiments described below. Cells were grown in liquid LB medium under aeration at 28 or 37°C. Antibiotics were added as recommended. Liquid culture growth was monitored as the OD_578_.

10.1128/mbio.03249-21.4 TABLE S1 Oligonucleotide primers used in the present study. Relevant nucleotides (e.g., restriction sites, mutations introduced or pKD13-specific sequences) are labeled in boldface. All primer sequences are given from the 5′ end to the 3′ end. Download Table S1, PDF file, 0.1 MB. Copyright © 2021 Junkermeier and Hengge. 2021 Junkermeier and Hengge. https://creativecommons.org/licenses/by/4.0/ This content is distributed under the terms of the Creative Commons Attribution 4.0 International license.

### Construction of the single copy *lacZ* reporter fusion.

The strain carrying the single copy *nfrB*::*lacZ* reporter fusion also carries a Δ(*lacI-A*)::*scar* deletion as previously described (5, 45). The primers used to construct the fusion are listed in Table S1. PCR fragments were cloned into the *lacZ* fusion vector pJL28, as previously described (45). The fusion was transferred to the att(λ) site of the chromosome via phage λRS45 (48). Single lysogeny was confirmed by PCR (49).

### Bacteriophages and propagation.

Phage N4 (GCA_000867865.1) was obtained from the Félix d’Hérelle Reference Center for Bacterial Viruses from the Université Laval (Quebec City, Quebec, Canada). The phage was propagated on E. coli K-12 strains using lysis on plates according to standard protocols (50). Phages were stored and diluted in SM buffer (100 mM NaCl, 8 mM MgSO_4_, 50 mM Tris-Cl) with 0.01% (wt/vol) gelatin.

### Protein purification.

NfrB^414-745^ was purified as a glutathione *S*-transferase-tagged fusion protein. The coding sequence was cloned on plasmid pGEX-6P-1 (Cytiva, catalog no. 58-9546-48) using the primers listed in Table S1. E. coli BL21 Gold strain was transformed with the plasmid and grown to an OD_578_ of 0.6 in LB medium at 28°C, when IPTG (0.1 mM) was added, and incubation proceeded for additional 4 h. Cells were harvested and resuspended in lysis buffer (140 mM NaCl, 2.7 mM KCl, 10 mM Na_2_HPO_4_ 1.8 mM KH_2_PO_4_, 5 mM dithiothreitol [DTT]; pH 7.3) containing protease inhibitor cocktail (complete, EDTA-free; Roche). Cells were disrupted by two passages through a French press. Insoluble material was removed by centrifugation. The supernatant was incubated under gentle shaking overnight with Glutathione-Sepharose 4B (Cytiva 17-0756-01; 1 mL per 1,000-mL cell culture) at 4°C. The resin was washed with cleavage buffer (50 mM Tris-Cl, 150 mM NaCl, 1 mM EDTA, 1 mM DTT; pH 7.0). PreScission protease (Cytiva, 27-0843-01; 80 μL of protease in 920 μL of binding buffer per bed volume) was added, followed by incubation at 4°C overnight to elute the protein.

Membrane-associated DgcJ^His^ and DgcQ^His^ were purified from IPTG-induced E. coli BL21 Gold cells, transformed with pRH800-DgcJ^His^ or pRH800-DgcQ^His^, respectively (pRH800 is a medium-copy-number p_tac_ expression vector). Cells were harvested by centrifugation and resuspended in lysis buffer 50 mM Tris (pH 8.0), 10 mM MgCl_2_ 300 mM NaCl, 1 mM EDTA, and protease inhibitor cocktail (complete, EDTA-free; Roche). Cells were disrupted by two passages through a French press. Intact cells were removed by centrifugation for 20 min at 5,000 rpm, and total membranes were collected by ultracentrifugation for 60 min at 36.000 rpm. The membrane pellet was solubilized in 50 mM Tris (pH 8.0), 10 mM MgCl_2_, 300 mM NaCl, 5% glycerol, and 2% dodecyl-β-d-maltoside (DDM; Roth) for 2 h at 4°C. Solubilized and nonsolubilized proteins were separated by ultracentrifugation. The supernatant was incubated with Ni-NTA Agarose (Qiagen) at 4°C. The resin was washed using solubilization buffer supplemented with 0.05% DDM. Proteins were eluted using solubilization buffer supplemented with 250 mM imidazole.

### Differential radial capillary action of ligand assay.

DRaCALA assays were performed using 20 μM purified NfrB^414-745^ incubated with 4 nM ^32^P-labeled c-di-GMP, as described previously (51). Radiolabeled nucleotides were obtained from Hartmann Analytic GmbH. Samples were spotted on nitrocellulose after a 10-min incubation at room temperature.

### Microscale thermophoresis.

NfrB^414-745^ was labeled by using a RED-NHS protein labeling kit (NanoTemper Technologies). The labeling reaction was performed according to the manufacturer’s instructions in the supplied labeling buffer, applying a concentration of 20 μM protein at room temperature for 30 min in the dark. Unreacted dye was removed with the supplied dye removal column equilibrated with MST buffer (137 mM NaCl, 2.7 mM KCl, 10 mM Na_2_HPO_4_, 1.8 mM KH_2_PO_4_, 0.05% Tween). The degree of labeling was determined using UV/VIS spectrophotometry at 650 and 280 nm. The labeled protein was adjusted to 80 nM with MST buffer. c-di-GMP and GTP was dissolved in MST buffer, and a series of 16 1:2 dilutions was prepared using the same buffer. For the measurement, each ligand dilution was mixed with 1 volume of labeled protein, which led to a final concentration of 40 nM and final ligand concentrations ranging from 0.00153 to 50 μM. After a 10-min incubation, the samples were loaded into Monolith NT.115 Premium Capillaries (NanoTemper Technologies). MST was measured using a Monolith NT.115 instrument (NanoTemper Technologies) at an ambient temperature of 25°C. Instrument parameters were adjusted to 20% LED power and 40% MST power. The data for three independently pipetted measurements were analyzed (MO.Affinity Analysis software version 2.3; NanoTemper Technologies).

### Determination of β-galactosidase activity.

The β-galactosidase activity was assayed by using *o*-nitrophenol galactoside (ONPG) as a substrate and is reported as μmol of *o*-nitrophenol min^−1^ (mg cellular protein)^−1^ (46). Experiments were performed at least twice, and the results from a representative experiment are shown. The OD_578_ was determined, and measurements were made as for the cells grown in liquid LB medium.

### Motility assay.

Bacterial motility was tested on swim plates containing 0.5 % Bacto tryptone, 0.5 % NaCl, and 0.3 % agar. A 3-μL volume of overnight culture (OD adjusted) was inoculated into the swim plates, and the cells were allowed to grow and swim for 4.5 h at 37°C.

### SDS-PAGE and immunoblot detection.

Proteins were detected by SDS polyacrylamide gel electrophoresis (SDS-PAGE) and immunoblotting as previously described (52) with antibodies against the Flag epitope (Sigma) or the 6×His tag (Bethyl Laboratories, Inc.) at a 1:10,000 dilution. Anti-rabbit or anti-mouse IgG-horseradish peroxidase conjugate from a donkey (GE Healthcare) was used (at a 1:20,000 dilution) for protein visualization in the presence of Western Lightning Plus-ECL enhanced chemiluminescence substrate (Perkin-Elmer). The WesternSure prestained chemiluminescent protein ladder (Li-Cor) was used as a molecular mass standard.

### Identification of NfrB homologs.

NfrB homologs were identified by using the E. coli NfrB protein as a query to perform TBLASTN searches of the NCBI nonredundant protein database. The data set was manually curated (Geneious software, Geneious Prime 2021.2.2) by removing false-positive hits, as well as disrupted operons (e.g., by mobile genetic elements or mutations in coding sequences). The tree was generated using phyloT (phyloT.biobyte.de) based on the NCBI taxonomy of the identified species, in which NfrB homologs were found. iTOL was used to generate the tree. A protein alignment of the 1,842 identified homologs was generated with a Genious Alignment (Blosom62 cost matrix, gap open penalty of 12, gap extension penalty of 3, and 2 refinement iterations).

### Additional software tools.

ImageJ (NIH) was used to calculate the swim diameters of motility plates and intensities of images of phosphorimager films of DRaCALA assays. Sequence logos were generated with the WebLogo service (https://weblogo.berkeley.edu/). Prism 9.2.0 (GraphPad Software, San Diego, CA) was used to generate graphs and for statistical analysis.
