# Supplementary figures and images for: A Novel Locally c-di-GMP-Controlled Exopolysaccharide Synthase Required for Bacteriophage N4 Infection of Escherichia coli
Source: mBio. 2021 Dec 14;12(6):e03249-21. doi: 10.1128/mbio.03249-21 (PMC8669469; doi:10.1128/mbio.03249-21)

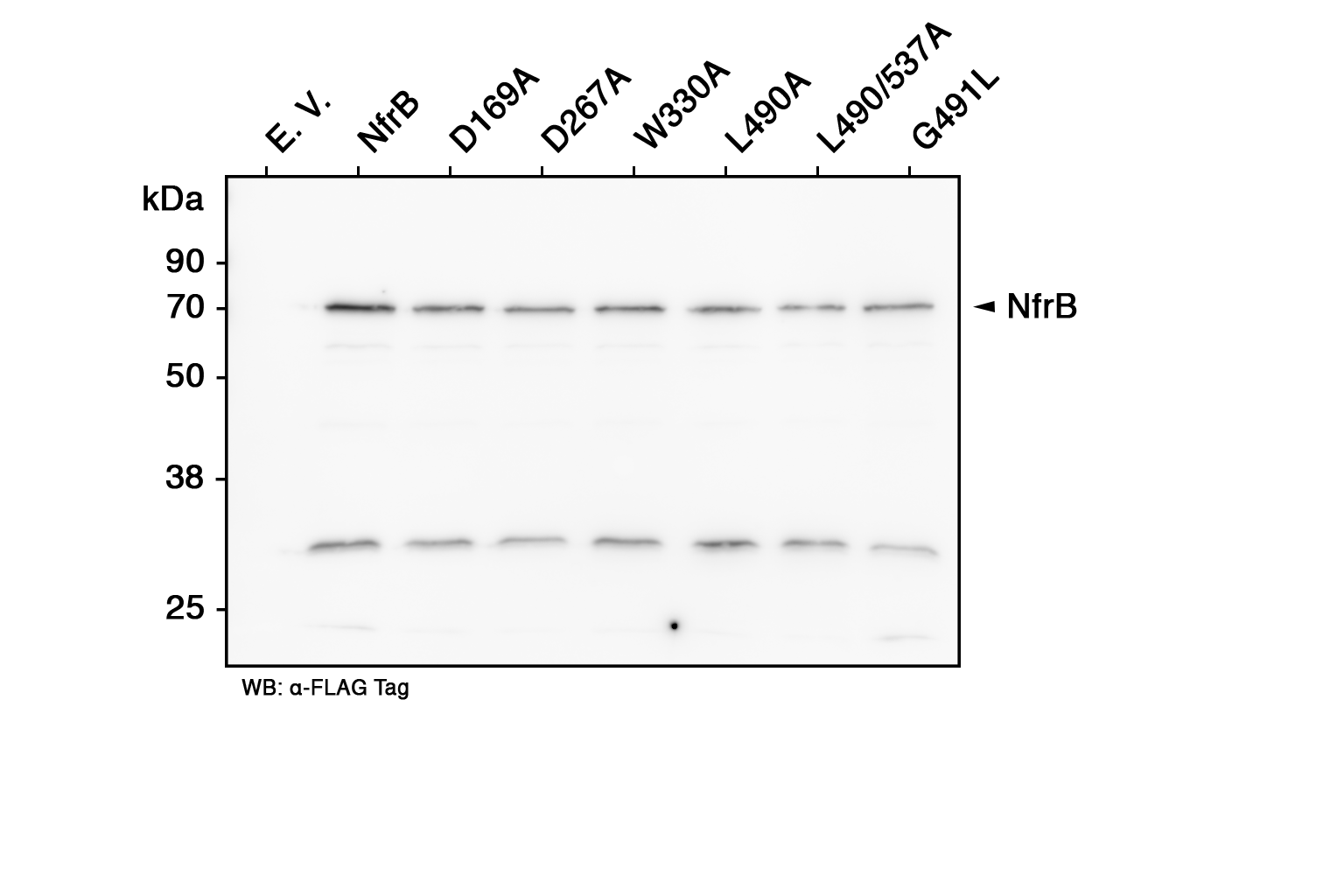

Supplement: FIG S2 [file mbio.03249-21-sf002.tif]

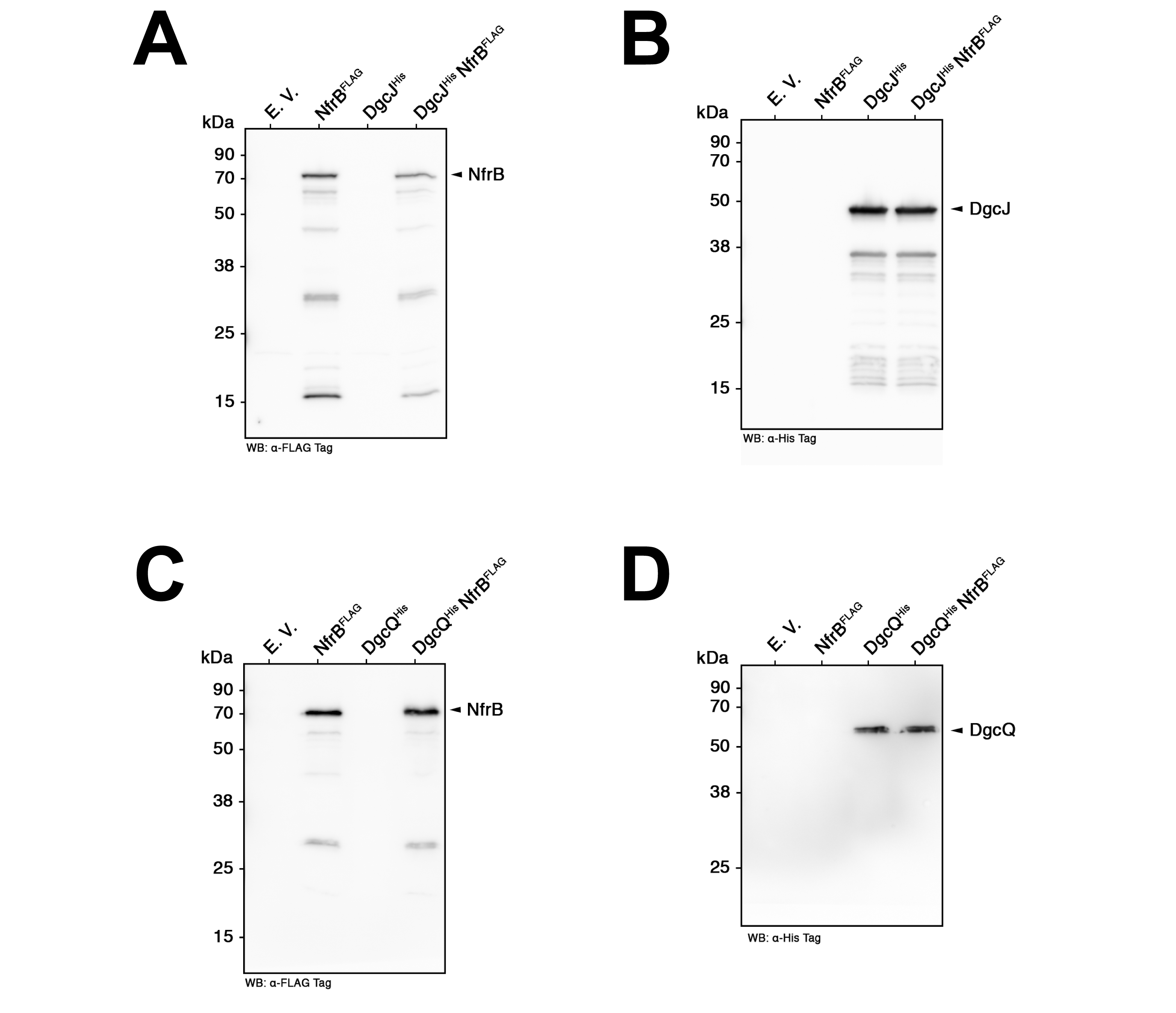

Supplement: FIG S3 [file mbio.03249-21-sf003.tif]
