# Supplementary material for: A Novel Locally c-di-GMP-Controlled Exopolysaccharide Synthase Required for Bacteriophage N4 Infection of Escherichia coli
Source: mBio. 2021 Dec 14;12(6):e03249-21. doi: 10.1128/mbio.03249-21 (PMC8669469; doi:10.1128/mbio.03249-21)
Supplement: TABLE S1 [file mbio.03249-21-st001.pdf]

Table S1

Oligonucleotide primers used in the present study[1].

I. Primers used for cloning *nfrB* into pGEX-6P-1 (Cytiva 58-9546-48):

NfrB$^{451-745}$_BamHI_f	cat**ggatcc**atggttaaccgcatcgtgcag
NfrB$^{451-745}$_EcoRI_r	gta**gaattc**ttattctccttcattttcggactc

II. Primers used for generating the *nfrB::lacZ* reporter gene fusion:

nfrBlacZ-1499_f	catatg**gaattc**gcatggatcatgatccactc
nfrBlacZ+0_r	gat**aagctt**ccatacaaaaaccttacattaacg

III. Primers used for generating chromosomal knockout mutations by one-step inactivation (OSI):

nfrB-H1P1	gccaccctaaacataaccagcgttaatgtaaggttttgt**gtgtaggctggagctgcttc**
nfrB-H2P2	aggttattctccttcattttcggactccagttgcgcaacc**attccggggatccgtcgacc**
nfrA-H1P1	acaggttgcgcaactggagtccgaaaatgaaggagaataa**gtgtaggctggagctgcttc**
nfrA-H2P2	gaaaatgaacttacgcatttaccagtgcactccaatggtg**attccggggatccgtcgacc**
ybcH-H1P1	gcgcaacaacgcgtttctcaccattggagtgcactggtaa**gtgtaggctggagctgcttc**
ybcH-H2P2	gaaagaggtaagccaggtcgtacccgacttacctggaggagat**attccggggatccgtcg acc**
wecA-G-H1P1	ggtcttcgtggttatacttctgctaataattttctctgagagcatgcattgt**gtaggctg gagctgcttc**
wecA-G-H2P2	gcagacaggcgacggagtgaccactccgtcgctttacaaagagaggaaaa**attccgggga tccgtcgacc**
wecB-H1P1	gagcgcaaaggcgctcgccgcttattcgaagagaatcgat**gtggtgtaggctggagctgc ttc**
wecB-H2P2	gaaatggtcgcaaaactcatagtgatatccgattatttttaacgc**attccggggatccg tcgacc**
wecC-H1P1	ctcgcattctggaagcgttaaaaaataatcggatatcactatga**gtgtaggctggagctg cttc**
wecC-H2P2	gttatcagaatttttctcatcagcgccagactcctttggcatcgac**attccggggatccg tcgacc**

IV. Primers used for generating the plasmid pAP58 by replacing the antibiotic resistance cassette of pCAB18 (Barembruch and Hengge, 2007) with the chloramphenicol resistance cassette of pACYC184 (Chang and Cohen, 1978):

pCAB18-BglII-rew	**ggcggc**agatct**gttgaatactcat**actcttcc
pCAB18-NdeI-for	ggcggc**catatg**ctgtcagaccaagtttactc
CAT-BglII-for	ggcggc**agatct**ggtgcttttgccgttacgcac
CAT-NdeI-rew	ggcggc**catatg**aataactgccttaaaaaaattac**gcc**

V. Primers used for cloning *nfrBA-ybcH* and mutant alleles into pAP58:

*nfrB*_XmaI_f	ct**cccggg**acataaccagcgttaatgtaag
*ybcH*_XbaI_r	ga**tctaga**ttaatactcgagaatgccgtg
nfrB^{L490A}_f	cgcccg**gcg**ggtcaaattc
nfrB^{L490A}_r	gaatttgacc**cgc**cgggcg
nfrB^{L537A}_f	gctggcacaggcg**gcg**gcagagcaaaac
nfrB^{L537A}_r	gttttgctctgc**cgc**cgcctgtgccagc
nfrB^{G491L}_f	gttgcgcccgtta**ctg**caaattctgctgg
nfrB^{G491L}_r	ccagcagaatttg**cag**taacgggcgcaac

[1] Relevant nucleotides (e.g. restriction sites, mutations introduced or pKD13-specific sequences) labeled in **bold**. All primer sequences are given from 5´- to 3´-end.

nfrB^{D169A}_f	attctgcat**gcc**gccgaagatgtgatttc
nfrB^{D169A}_r	gaaatcacatcttcggc**ggc**atgcagaat
nfrB^{D267A}_f	gagtcttactgaa**gcg**tacgacattggcttc
nfrB^{D267A}_r	gaagccaatgtcgta**cgc**ttcagtaagactc
nfrB^{W330A}_f	atccc**gcg**cgatcatcggcattgttttc
nfrB^{W330A}_r	gaaaacaatgccgatgatcg**cgc**gggat
*nfrA*_BamHI_r	gta**ggatcc**gatcgtccagctg
nfrB_ClaI_rev	catt**atcgat**ggattcccacgcc

VI. Primers used for cloning *dgcJ* and *dgcQ* into pRH800 (Lange and Hengge-Aronis, 1994) and generating mutant alleles:

*dgcJ*_BamHI_f	cat**ggatcc**ctcgtttcactaaccgaagg
*dgcJ*_XbaI_r	gt**tctaga**tcatgaacggctgtttttgttc
dgcJ^{6xHIS}_XbaI_r	gt**tctaga**tcagtgatggtgatggtgatgtgaacggctgtttttgttc
dgcJ^{GGAAF}_f	ctcggtggc**gctgca**ttctgcatc
dgcJ^{GGAAF}_r	gatgcagaa**tgcagc**gccaccgag
*dgcQ*_BamHI_f	cata**ggatcc**gaatcataaaaaagcaggttggg
dgcQ^{6xHIS}_XbaI_r	at**tctaga**ttagtgatggtgatggtgatgagcgttatcgctcgcga

VII. Primers used for cloning *nfrB*^{3xFLAG} into pAP58:

NfrB^{3xFLAG}*_f*	cacgacatcgactacaaggacgacgacgacaag**caactggagtccgaaaatg**
NfrB^{3xFLAG}*_r*	gtccttgtagtcaccgtcgtggtccttgtagtc**cgcaacctgttctgtgttta**

VIII. Primers used for chromosomal C-terminal 3xFLAG-tagging of *nfrB* via two-step mutagenesis:

nfrB_ccdB_f	tcgttcagcaattaacgtgttgttattgcgccatgaacgcagttctctgccgctcggcaa **cgcatcgtggccggatcttgc**
nfrB_ccdB_r	ggcgttctgccgcgcttaatgtgttctgctggcgtgcttcattgtatagcgtatctgcct **cggataacagaaaggccggg**
nfrB^{3xFLAG}*_f*	tgccgcatcagttcctgttc
nfrB^{3xFLAG}*_r*	cagtgccaggatccgatcgt
